# The genome sequences of the diplonemid protist *Diplonema japonicum* YPF1604 and its bacterial endosymbionts *Ca.* Cytomitobacter primus and *Ca.* Nesciobacter abundans

**DOI:** 10.12688/wellcomeopenres.23917.1

**Published:** 2025-04-14

**Authors:** Daria Tashyreva, Drahomíra Faktorová, Eva Stříbrná, Aleš Horák, Julius Lukeš, John M. Archibald, Graeme Oatley, Elizabeth Sinclair, Eerik Aunin, Noah Gettle, Camilla Santos, Michael Paulini, Haoyu Niu, Victoria McKenna, Rebecca O’Brien, Wellcome Sanger Institute Tree of Life Management, Samples and Laboratory Team, Wellcome Sanger Institute Scientific Operations: Sequencing Operations, Wellcome Sanger Institute Tree of Life Core Informatics Team, EBI Aquatic Symbiosis Genomics Data Portal Team, Aquatic Symbiosis Genomics Project Leadership

**Affiliations:** 1 Institute of Parasitology, Biology Centre, České Budějovice, Czech Republic; 2 Department of Biochemistry and Molecular Biology, Dalhousie University, Halifax, Nova Scotia, Canada; 3 Tree of Life, Wellcome Sanger Institute, Hinxton, England, UK

**Keywords:** Diplonema japonicum, diplonemid, genome sequence, chromosomal, Euglenozoa

## Abstract

We present a genome assembly of the diplonemid *Diplonema japonicum* YPF1604 (Discoba; Euglenozoa; Diplonemea; Diplonemidae). The genome sequence is 62.30 megabases in span. Most of the assembly is scaffolded into 118 chromosomal pseudomolecules. The multipartite mitochondrial genome was also assembled. The genome sequences of two bacterial endosymbionts, *Ca*. Cytomitobacter primus and *Ca.* Cytomitobacter primus, were also assembled.

## Species taxonomy (host)

Eukaryota; Discoba; Euglenozoa; Diplonemea; Diplonemidae; *Diplonema*; *Diplonema japonicum* Tashyreva *et al.*, 2018 (NCBI:txid2508216).

## Species taxonomy: endosymbionts

### *Ca.* Cytomitobacter primus

Bacteria; Pseudomonadota; Alphaproteobacteria; Rhodospirillales; Holosporaceae; Candidatus Cytomitobacter/Nesciobacter (NCBI:txid2066024)

### *Ca.* Nesciobacter abundans

Bacteria; Pseudomonadota; Alphaproteobacteria; Holosporales; Holosporaceae; Candidatus Nesciobacter (NCBI:txid2601668)

## Background

*Diplonema japonicum* YPF1604 was isolated from the sand filter of ‘A-14’ tank in Enoshima Aquarium (Fujisawa, Kanagawa, Japan). This representative of the family Diplonemidae, or ‘classic diplonemids’, has a complex life cycle comprised of three distinct life stages. A large gliding trophic stage (~20 μm long) with short flagella is characteristic of growth under nutrient-rich conditions. Following the depletion of nutrients, the organism transforms into a sessile stage and subsequently into a small fast-swimming stage with long flagella. The latter two stages are characterised by the presence of tubular extrusomes and the emergence of a paraflagellar rod, the supportive structure of the flagellum, which is prominently lacking in the trophic stage.

We identified the presence of two species of endosymbionts, *Ca.* Cytomitobacter primus and *Ca.* Nesciobacter abundans in the cytoplasm of *D. japonicum* ( George *et al.*, 2020; Tashyreva *et al.*, 2018). These species belong to closely related genera that form a novel lineage within Holosporaceae, a family of Alphaproteobacteria with exclusively endosymbiotic lifestyle ( Muñoz-Gómez *et al.*, 2019). In total, this lineage includes five distinct *Candidatus* species of Cytomitobacter ( George *et al.*, 2020; our unpublished data) and one species of *Ca.* Nesciobacter, all reported only from the diplonemid hosts. Both *C. primus* and *N. abundans* closely interact with the mitochondrion, with bacterial cells often entirely surrounded by the organelle. This intimate relationship is attributed to the symbionts’ inability to generate ATP through substrate or oxidative phosphorylation, thus designating them as so-called energy parasites. All the symbionts are equipped with a wide arsenal of effector and secretion systems, which are believed to manipulate the hosts’ metabolism ( George *et al.*, 2020). Although the symbionts’ genomes had been previously sequenced and analysed, genome sequences from the host generated here will be instrumental in identifying the potential targets for bacterial effectors and uncovering mechanisms of bacterial interactions with the host mitochondria.

## Genome sequence report

**Figure 1.**
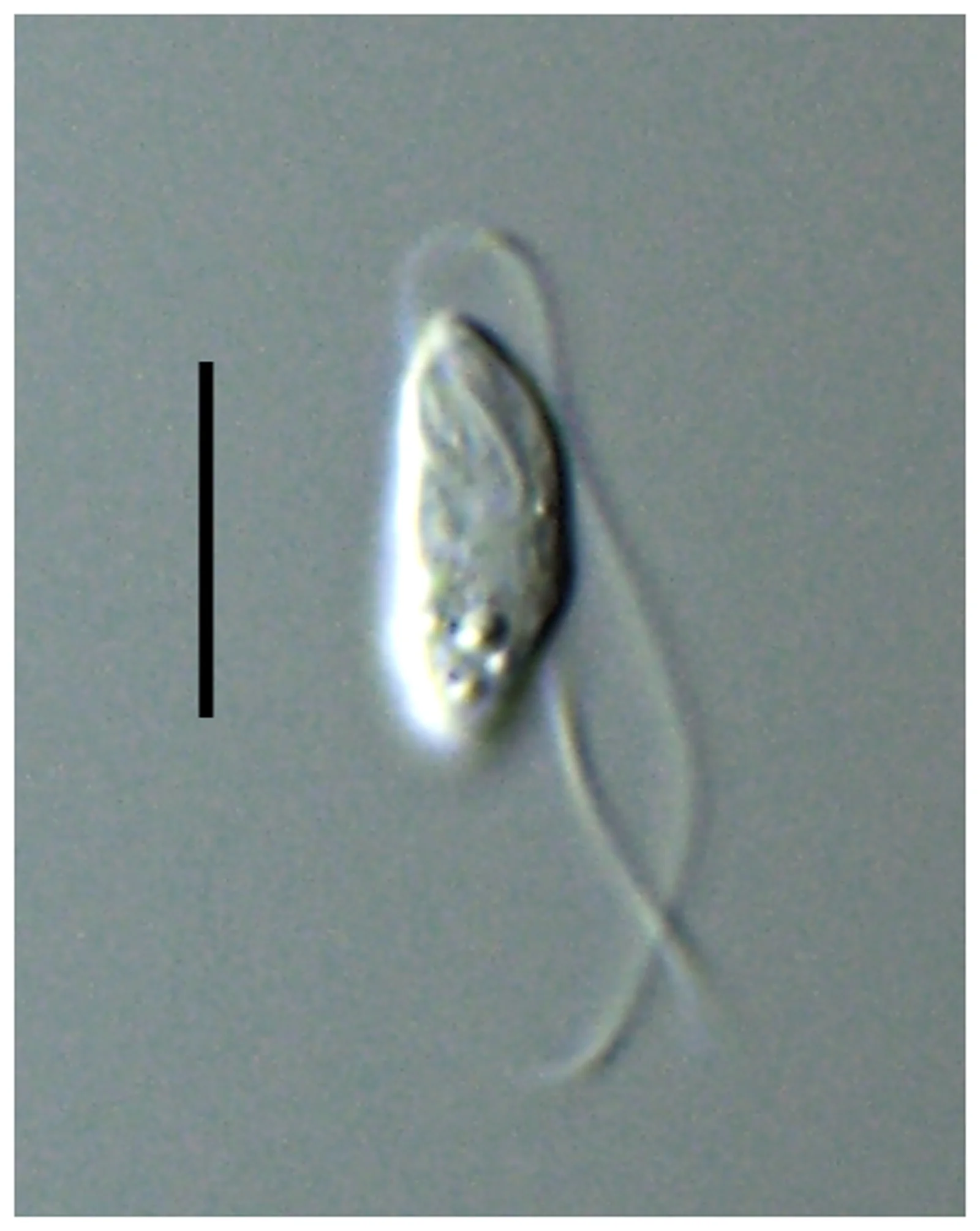
Differential interference contrast micrograph of *Diplonema japonicum* (peDipJapo1) in the swimming stage. Scale bar is 10 μm.

The nuclear genome was sequenced from cells of *Diplonema japonicum* ( Figure 1) cultured at the Biology Centre, Czech Republic. A total of 102-fold coverage in Pacific Biosciences single-molecule HiFi long reads was generated. Primary assembly contigs were scaffolded with chromosome conformation Hi-C data. Manual assembly curation corrected 69 missing joins or mis-joins and removed 6 haplotypic duplications, increasing the scaffold number by 0.76%, and decreasing the scaffold N50 by 1.83%.

**Figure 2.**
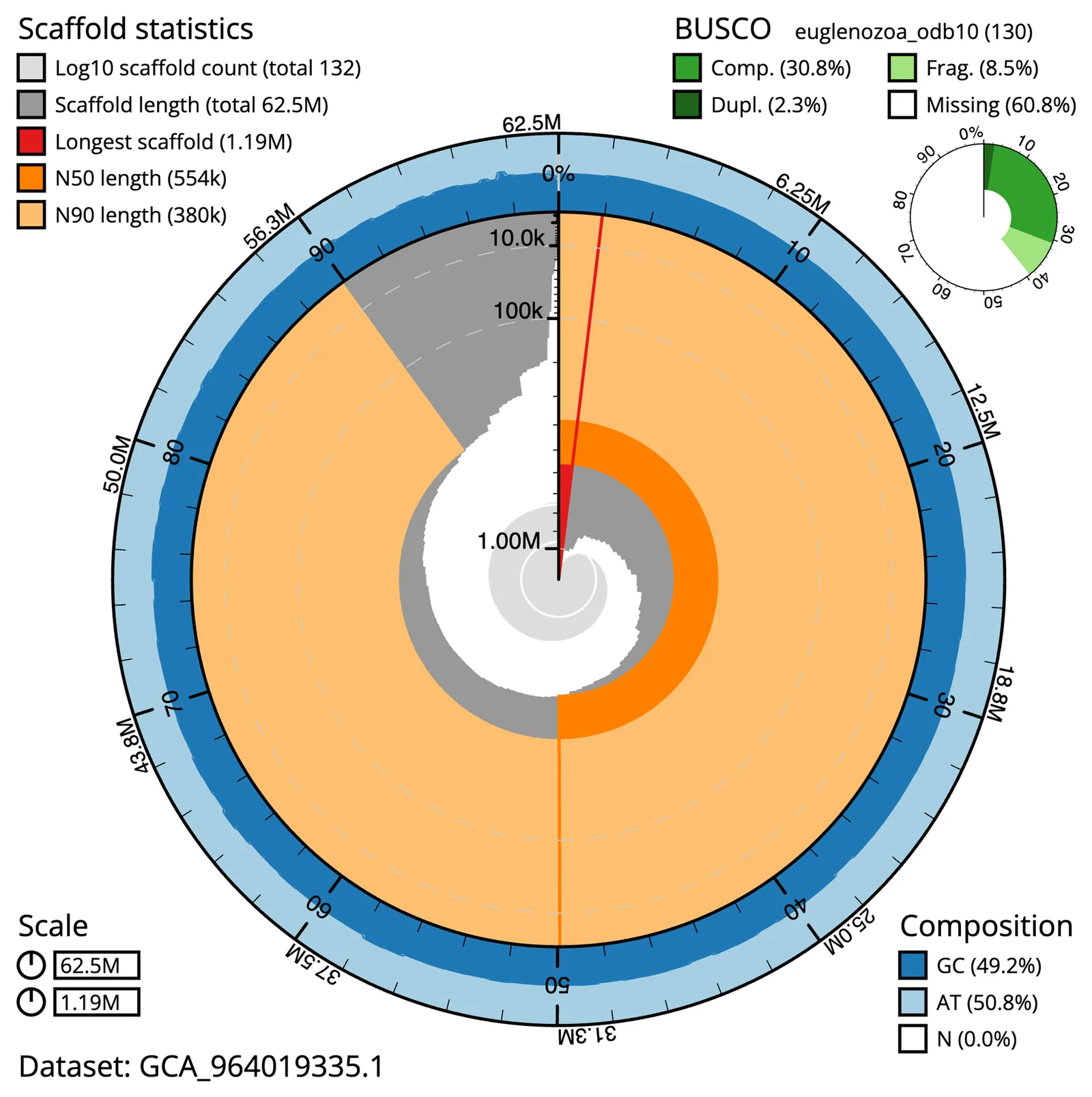
Genome assembly of *Diplonema japonicum*, peDipJapo1.1: metrics. The BlobToolKit snail plot shows N50 metrics and BUSCO gene completeness. The main plot is divided into 1,000 bins around the circumference with each bin representing 0.1% of the 62,535,345 bp assembly. The distribution of scaffold lengths is shown in dark grey with the plot radius scaled to the longest scaffold present in the assembly (1,191,308 bp, shown in red). Orange and pale-orange arcs show the N50 and N90 scaffold lengths (553,867 and 380,443 bp), respectively. The pale grey spiral shows the cumulative scaffold count on a log scale with white scale lines showing successive orders of magnitude. The blue and pale-blue area around the outside of the plot shows the distribution of GC, AT and N percentages in the same bins as the inner plot. A summary of complete, fragmented, duplicated and missing BUSCO genes in the euglenozoa_odb10 set is shown in the top right. An interactive version of this figure is available at https://blobtoolkit.genomehubs.org/view/Diplonema_japonicum/dataset/GCA_964019335.1/snail.

**Figure 3.**
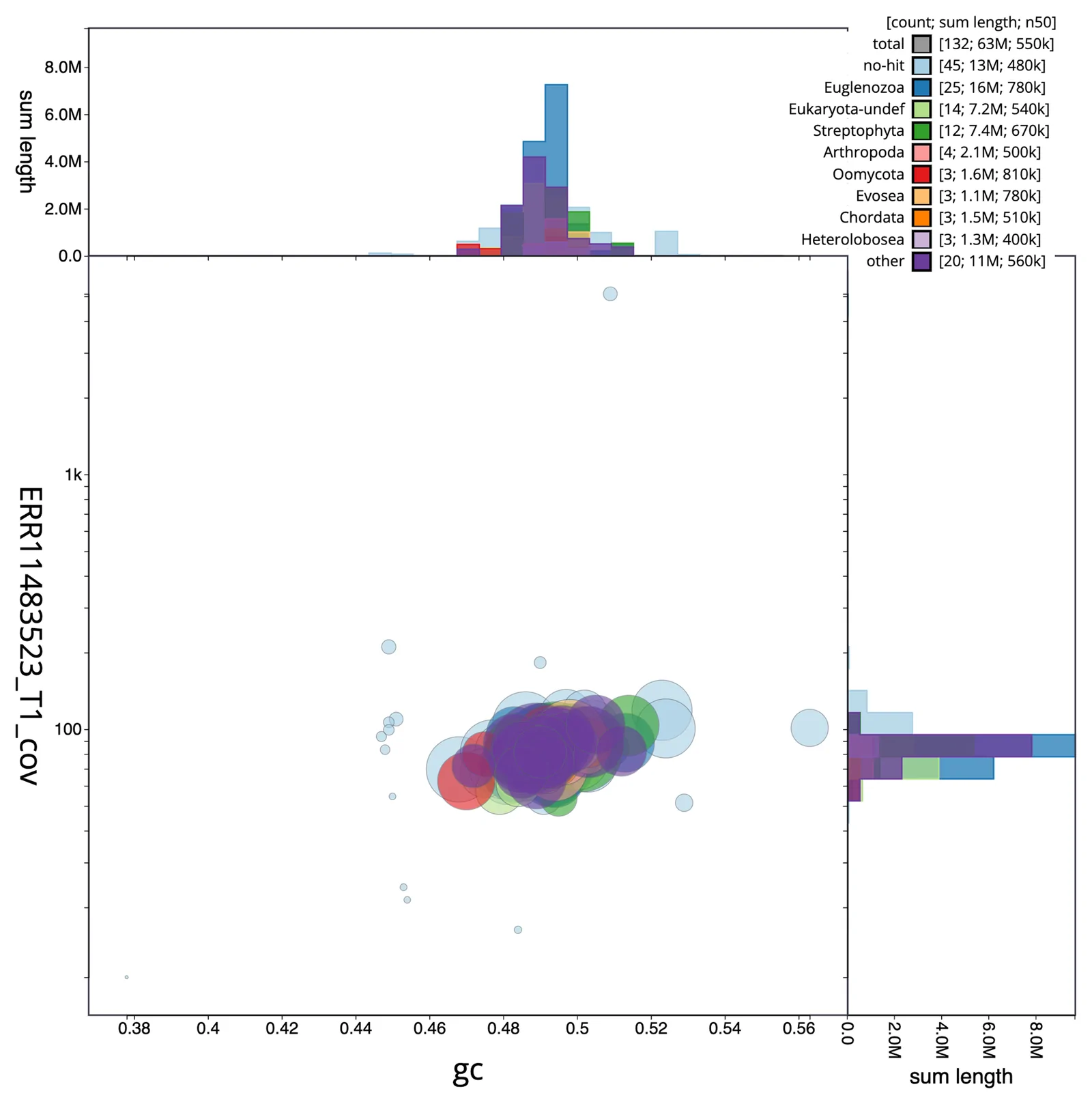
Genome assembly of *Diplonema japonicum*, peDipJapo1.1: BlobToolKit GC-coverage plot. Scaffolds are coloured by phylum. Circles are sized in proportion to scaffold length. Histograms show the distribution of scaffold length sum along each axis. An interactive version of this figure is available at https://blobtoolkit.genomehubs.org/view/Diplonema_japonicum/dataset/GCA_964019335.1/blob.

**Figure 4.**
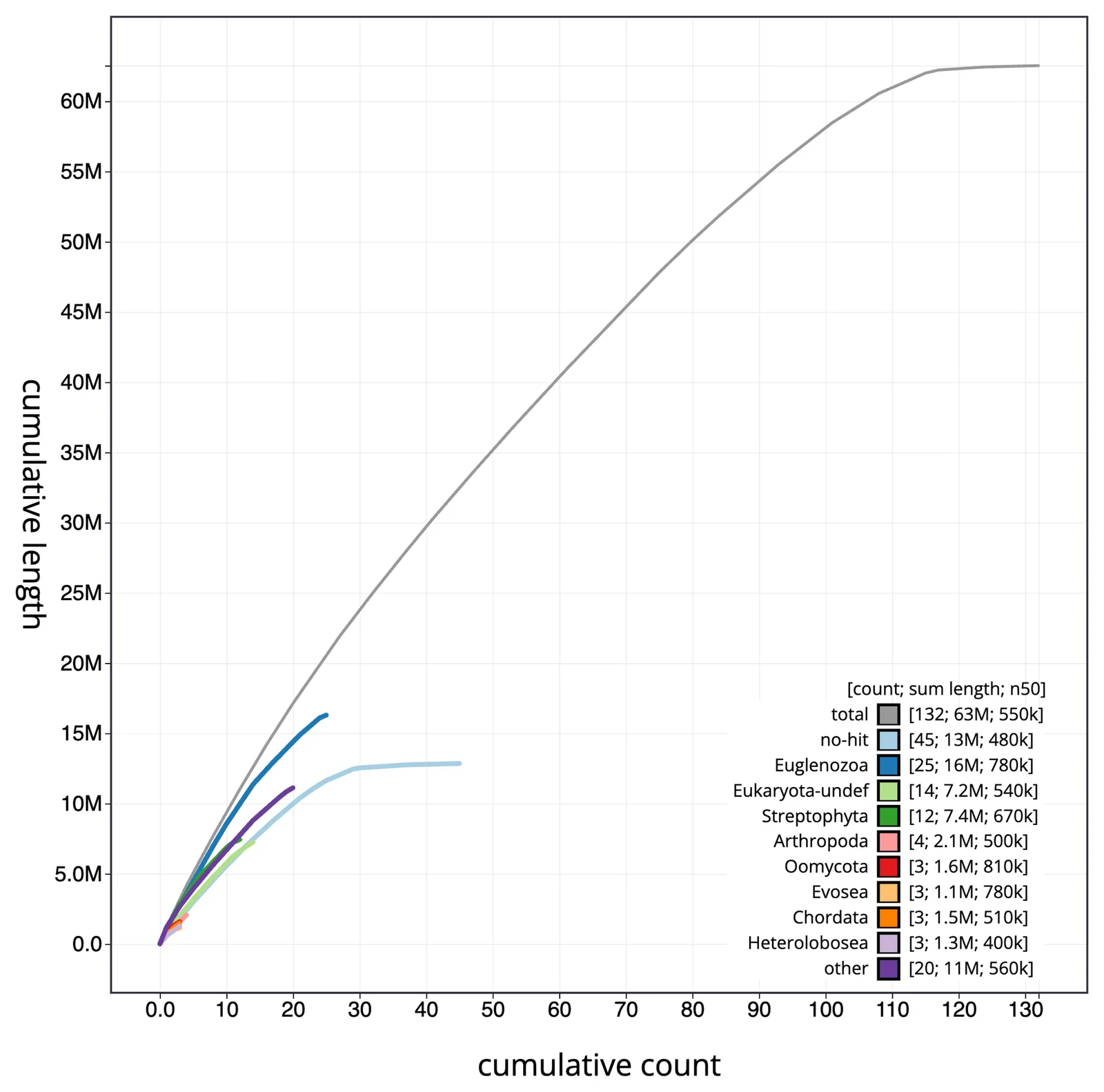
Genome assembly of *Diplonema japonicum*, peDipJapo1.1: BlobToolKit cumulative sequence plot. The grey line shows cumulative length for all scaffolds. Coloured lines show cumulative lengths of scaffolds assigned to each phylum using the buscogenes taxrule. An interactive version of this figure is available at https://blobtoolkit.genomehubs.org/view/Diplonema_japonicum/dataset/GCA_964019335.1/cumulative.

**Figure 5.**
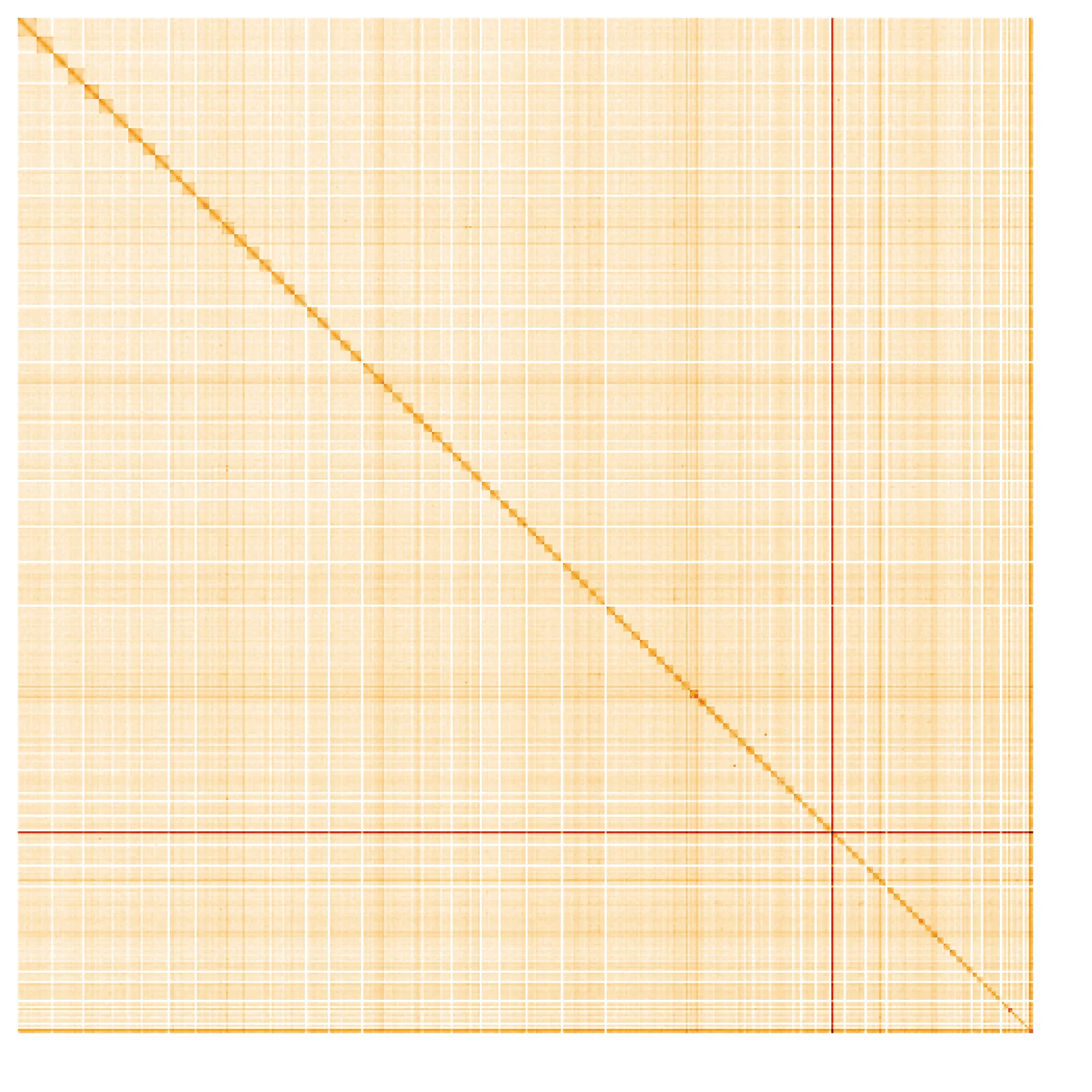
Genome assembly of *Diplonema japonicum*, peDipJapo1.1: Hi-C contact map of the peDipJapo1.1 assembly, visualised using HiGlass. Chromosomes are shown in order of size from left to right and top to bottom. An interactive version of this figure may be viewed at https://genome-note-higlass.tol.sanger.ac.uk/l/?d=CCl_vofbQY-R5WxJILzBkw.

The final nuclear genome assembly has a total length of 62.30 Mb in 121 sequence scaffolds with a scaffold N50 of 0.6 Mb ( Table 1). The snail plot in Figure 2 provides a summary of the assembly statistics, while the distribution of assembly scaffolds on GC proportion and coverage is shown in Figure 3. The cumulative assembly plot in Figure 4 shows curves for subsets of scaffolds assigned to different phyla. Most (99.58%) of the assembly sequence was assigned to 118 chromosomal-level scaffolds. Chromosome-scale scaffolds confirmed by the Hi-C data are named in order of size ( Figure 5; Table 2). While not fully phased, the assembly deposited is of one haplotype. Contigs corresponding to the second haplotype have also been deposited. The mitochondrial genome was also assembled and can be found as a contig within the multifasta file of the genome submission.

**Table 1.** Genome data for *Diplonema japonicum*, peDipJapo1.1.

Project accession data			
Assembly identifier	peDipJapo1.1		
Species	*Diplonema japonicum*		
Specimen	peDipJapo1		
NCBI taxonomy ID	2508216		
BioProject	PRJEB62625		
BioSample ID	Genome sequencing: SAMEA9312397 Hi-C scaffolding: SAMEA9312398 RNA sequencing: SAMEA9312400		
Isolate information	peDipJapo1 (genome sequence, Hi-C sequencing and RNA sequencing)		
Assembly metrics			
Consensus quality (QV)	59.4		
*k*-mer completeness	100.0%		
BUSCO *	C:30.8%[S:28.5%,D:2.3%],F:8.5%,M:60.7%,n:130		
Percentage of assembly mapped to chromosomes	99.58%		
Organelles	Mitochondrial genome: multipartite		
Sequencing information			
**Platform**	**Run accession**	**Read count**	**Base count (Gb)**
**Hi-C Illumina NovaSeq 6000**	ERR12765141	6.91e+08	104.33
**Hi-C Illumina NovaSeq X**	ERR12765143	8.84e+08	133.54
**PacBio Sequel IIe**	ERR11483523	8.40e+05	7.93
**RNA Illumina NovaSeq 6000**	ERR12245577	6.48e+07	9.79
Genome assembly			
Assembly accession	GCA_964019335		
*Accession of alternate haplotype*	GCA_964019325.1		
Span (Mb)	62.30		
Number of contigs	242		
Contig N50 length (Mb)	0.4		
Number of scaffolds	121		
Scaffold N50 length (Mb)	0.6		
Longest scaffold (Mb)	1.19		

* BUSCO scores based on the euglenozoa_odb10 BUSCO set using version 5.4.3. C = complete [S = single copy, D = duplicated], F = fragmented, M = missing, n = number of orthologues in comparison. A full set of BUSCO scores is available at https://blobtoolkit.genomehubs.org/view/Diplonema_japonicum/dataset/GCA_964019335.1/busco.

**Table 2.** Chromosomal pseudomolecules in the genome assembly of *Diplonema japonicum*, peDipJapo1.

INSDC accession	Name	Length (Mb)	GC%
OZ025648.1	1	1.19	49.0
OZ025649.1	2	1.0	50.0
OZ025650.1	3	1.0	49.0
OZ025651.1	4	0.98	49.5
OZ025652.1	5	0.9	50.0
OZ025653.1	6	0.88	49.0
OZ025654.1	7	0.87	49.0
OZ025655.1	8	0.86	50.0
OZ025656.1	9	0.85	49.5
OZ025657.1	10	0.83	49.5
OZ025658.1	11	0.82	49.5
OZ025659.1	12	0.82	49.0
OZ025660.1	13	0.81	49.5
OZ025661.1	14	0.8	49.0
OZ025662.1	15	0.78	50.0
OZ025663.1	16	0.78	49.5
OZ025664.1	17	0.76	49.5
OZ025665.1	18	0.73	50.5
OZ025666.1	19	0.73	49.5
OZ025667.1	20	0.73	49.5
OZ025668.1	21	0.7	48.5
OZ025669.1	22	0.7	49.5
OZ025670.1	23	0.7	49.5
OZ025671.1	24	0.68	49.5
OZ025672.1	25	0.68	49.0
OZ025673.1	26	0.67	48.5
OZ025674.1	27	0.64	48.5
OZ025675.1	28	0.63	48.5
OZ025676.1	29	0.62	48.5
OZ025677.1	30	0.62	47.0
OZ025678.1	31	0.62	49.5
OZ025679.1	32	0.61	49.5
OZ025680.1	33	0.6	49.5
OZ025681.1	34	0.6	48.5
OZ025682.1	35	0.59	50.0
OZ025683.1	36	0.59	49.5
OZ025684.1	37	0.59	47.5
OZ025685.1	38	0.58	49.5
OZ025686.1	39	0.58	49.5
OZ025687.1	40	0.57	49.0
OZ025688.1	41	0.57	48.5
OZ025689.1	42	0.56	49.5
OZ025690.1	43	0.55	49.0
OZ025691.1	44	0.55	49.0
OZ025692.1	45	0.54	48.5
OZ025693.1	46	0.54	49.5
OZ025694.1	47	0.54	48.5
OZ025695.1	48	0.54	49.0
OZ025696.1	49	0.54	49.5
OZ025697.1	50	0.54	49.0
OZ025698.1	51	0.53	51.5
OZ025699.1	52	0.53	52.5
OZ025700.1	53	0.52	48.5
OZ025701.1	54	0.52	48.5
OZ025702.1	55	0.52	49.0
OZ025703.1	56	0.52	48.5
OZ025704.1	57	0.51	49.5
OZ025705.1	58	0.51	48.5
OZ025706.1	59	0.51	48.5
OZ025707.1	60	0.51	50.5
OZ025708.1	61	0.51	52.5
OZ025709.1	62	0.51	49.0
OZ025710.1	63	0.5	50.5
OZ025711.1	64	0.5	51.5
OZ025712.1	65	0.5	49.0
OZ025713.1	66	0.5	49.5
OZ025714.1	67	0.5	49.0
OZ025715.1	68	0.5	49.5
OZ025716.1	69	0.49	49.5
OZ025717.1	70	0.49	49.5
OZ025718.1	71	0.49	48.5
OZ025719.1	72	0.49	48.5
OZ025720.1	73	0.48	47.0
OZ025721.1	74	0.48	50.0
OZ025722.1	75	0.48	50.5
OZ025723.1	76	0.47	49.5
OZ025724.1	77	0.46	48.0
OZ025725.1	78	0.46	49.5
OZ025726.1	79	0.46	48.5
OZ025727.1	80	0.45	48.5
OZ025728.1	81	0.44	50.0
OZ025729.1	82	0.44	49.0
OZ025730.1	83	0.43	49.0
OZ025731.1	84	0.43	48.0
OZ025732.1	85	0.42	50.5
OZ025733.1	86	0.42	48.0
OZ025734.1	87	0.42	49.0
OZ025735.1	88	0.41	48.5
OZ025736.1	89	0.41	49.0
OZ025737.1	90	0.41	49.0
OZ025738.1	91	0.4	49.5
OZ025739.1	92	0.4	48.5
OZ025740.1	93	0.4	48.5
OZ025741.1	94	0.39	49.5
OZ025742.1	95	0.38	49.0
OZ025743.1	96	0.37	51.0
OZ025744.1	97	0.37	49.0
OZ025745.1	98	0.37	49.5
OZ025746.1	99	0.36	50.5
OZ025747.1	100	0.36	47.5
OZ025748.1	101	0.34	48.0
OZ025749.1	102	0.32	50.0
OZ025750.1	103	0.32	50.0
OZ025751.1	104	0.3	47.5
OZ025752.1	105	0.3	50.0
OZ025753.1	106	0.29	50.0
OZ025754.1	107	0.28	48.5
OZ025755.1	108	0.28	47.0
OZ025756.1	109	0.22	50.0
OZ025757.1	110	0.22	47.5
OZ025758.1	111	0.21	56.5
OZ025759.1	112	0.21	51.0
OZ025760.1	113	0.21	49.5
OZ025761.1	114	0.19	49.0
OZ025762.1	115	0.19	50.0
OZ025763.1	116	0.14	49.5
OZ025764.1	117	0.09	48.0
OZ025765.1	118	0.05	53.0
OZ025766.1	MT1	0.02	51.0
OZ025775.1	MT10	0.01	48.5
OZ025776.1	MT11	0.02	45.0
OZ025767.1	MT2	0.03	49.0
OZ025768.1	MT3	0.02	45.0
OZ025769.1	MT4	0.01	45.5
OZ025770.1	MT5	0.03	45.0
OZ025771.1	MT6	0.01	45.5
OZ025772.1	MT7	0.02	45.0
OZ025773.1	MT8	0.02	45.0
OZ025774.1	MT9	0.03	45.0

The primary assembly has a BUSCO v5.4.3 completeness of 30.8% (single = 28.5%, duplicated = 2.3%), using the euglenozoa_odb10 reference set ( *n* = 130). The low BUSCO coverage may be attributed to factors such as a poor orthologue lineage set for Euglenozoa, with inadequate representation of diplonemids. Additionally, the gene-finding algorithms in BUSCO may not identify gene features accurately in diplonemids. The estimated Quality Value (QV) of the final nuclear assembly is 59.4, and the *k*-mer completeness of the primary and alternate haplotypes is 90.32%.

**Figure 6.**
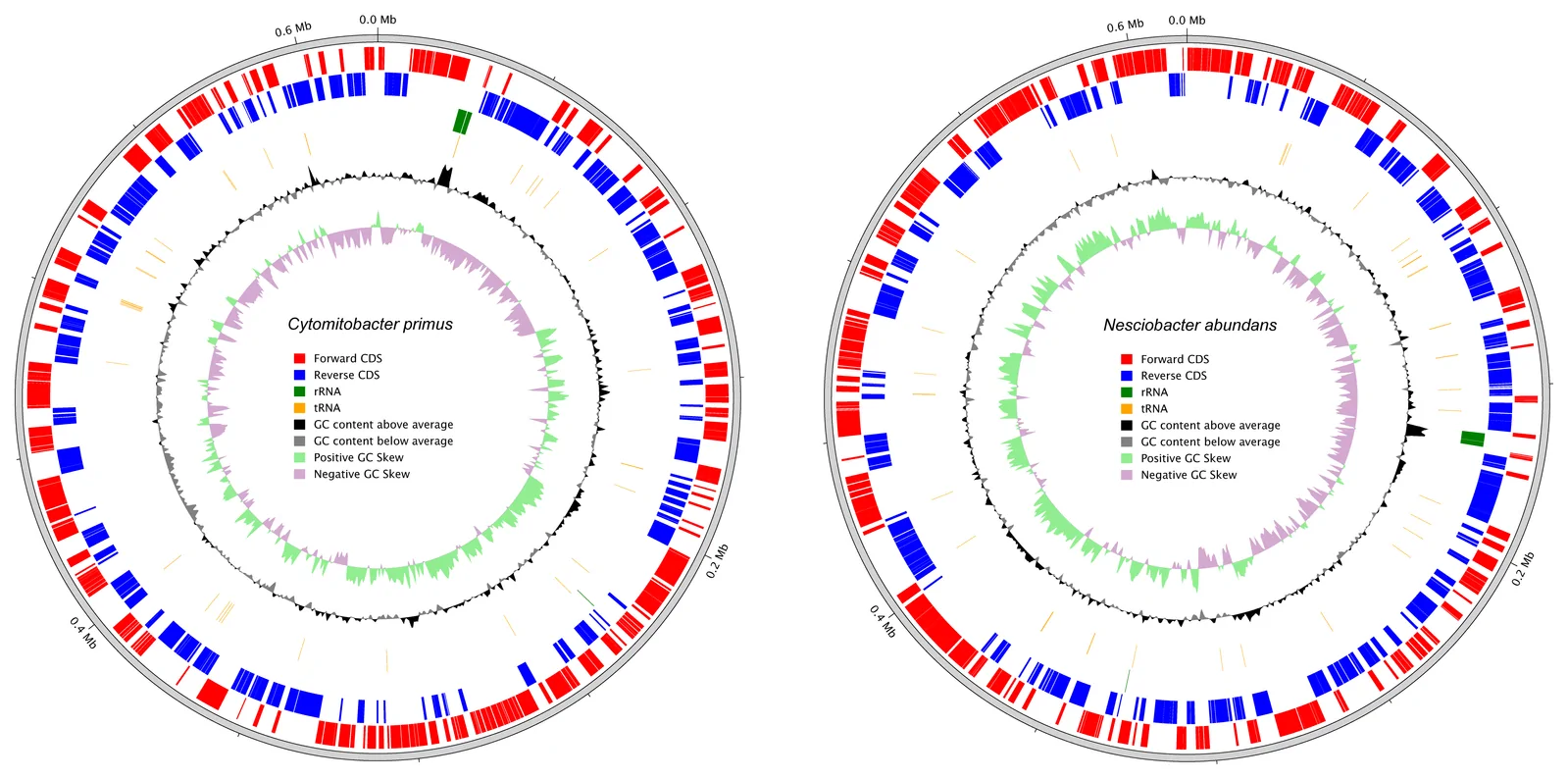
Genome maps of the assembled endosymbiont genomes *Ca.* Cytomitobacter primus and *Ca.* Nesciobacter abundans.

The genomes of two bacterial endosymbionts were also assembled ( Table 3; Figure 6).

**Table 3.** Assemblies of endosymbionts.

BioProject	Title	Assembly accession	Assembly length (kb)
PRJEB62716	*Candidatus* Cytomitobacter primus genome assembly, peDipJapo1.Cytomitobacter_primus_1	GCA_951805245.1	622.38
PRJEB64525	*Candidatus* Nesciobacter abundans genome assembly, peDipJapo1.Nesciobacter_abundans_1	GCA_960610765.1	616.06

## Methods

### Sample acquisition

*Diplonema japonicum* YPF1604 (specimen ID DU0000009, ToLID peDipJapo1) cells were grown axenically in seawater-based Hemi medium supplemented with 1% v/v horse blood serum at the Institute of Parasitology, Biology Centre, Branišovská, Czech Republic. Cells were collected on 2021-04-12 by centrifugation. The sample was collected and identified by Daria Tashyreva (Institute of Parasitology, Biology Centre) and preserved by snap-freezing the pellet.

### Nucleic acid extraction

The workflow for high molecular weight (HMW) DNA extraction at the Wellcome Sanger Institute (WSI) Tree of Life Core Laboratory includes a sequence of core procedures: sample preparation, sample homogenisation, DNA extraction, fragmentation, and clean-up. In sample preparation, the peDipJapo1 sample was weighed and dissected on dry ice ( Jay *et al.*, 2023). The cell pellet was homogenised using a PowerMasher II tissue disruptor ( Denton *et al.*, 2023a).

HMW DNA was extracted using the Automated MagAttract v1 protocol ( Sheerin *et al.*, 2023). DNA was sheared into an average fragment size of 12–20 kb in a Megaruptor 3 system with speed setting 30 ( Todorovic *et al.*, 2023). Sheared DNA was purified by solid-phase reversible immobilisation ( Strickland *et al.*, 2023), using AMPure PB beads t to eliminate shorter fragments and concentrate the DNA. The concentration of the sheared and purified DNA was assessed using a Nanodrop spectrophotometer, Qubit Fluorometer and Qubit dsDNA High Sensitivity Assay kit. Fragment size distribution was evaluated by running the sample on the FemtoPulse system.

RNA was extracted from peDipJapo1 cells in the Tree of Life Laboratory at the WSI using the RNA Extraction: Automated MagMax™ *mir*Vana protocol ( do Amaral *et al.*, 2023). The RNA concentration was assessed using a Nanodrop spectrophotometer and a Qubit Fluorometer using the Qubit RNA Broad-Range Assay kit. Analysis of the integrity of the RNA was done using the Agilent RNA 6000 Pico Kit and Eukaryotic Total RNA assay.

Protocols developed by the WSI Tree of Life Laboratory are publicly available on protocols.io ( Denton *et al.*, 2023b).

### Sequencing

Pacific Biosciences HiFi circular consensus DNA sequencing libraries were constructed according to the manufacturers’ instructions. Poly(A) RNA-Seq libraries were constructed using the NEB Ultra II RNA Library Prep kit. DNA and RNA sequencing was performed by the Scientific Operations core at the WSI on Pacific Biosciences Sequel IIe (HiFi) and Illumina NovaSeq 6000 (RNA-Seq) instruments. Hi-C data were also generated from cells of peDipJapo1 using the Arima2 kit and sequenced on the Illumina NovaSeq X instrument.

### Genome assembly and curation

Assembly of the host genome was carried out with Hifiasm ( Cheng *et al.*, 2021) and haplotypic duplication was identified and removed with purge_dups ( Guan *et al.*, 2020). The assembly was then scaffolded with Hi-C data ( Rao *et al.*, 2014) using YaHS ( Zhou *et al.*, 2023). The assembly was checked for contamination and corrected using the TreeVal pipeline ( Pointon *et al.*, 2023). Manual curation pf the host genome assembly was performed using JBrowse2 ( Diesh *et al.*, 2023), HiGlass ( Kerpedjiev *et al.*, 2018) and Pretext ( Harry, 2022).

The mitochondrial genome was assembled using MitoHiFi ( Uliano-Silva *et al.*, 2023), which runs MitoFinder ( Allio *et al.*, 2020) and uses these annotations to select the final mitochondrial contig and to ensure the general quality of the sequence.

The bacterial cobiont assemblies were generated using metaMDBG ( Benoit *et al.*, 2024) and identified using GTDB-TK (version 2.3.2; GTDB (release 214)) ( Chaumeil *et al.*, 2022). PROKKA (version 1.14.5) ( Seemann, 2014) was used to identify tRNAs and rRNAs and CheckM (version 1.2.1; checkM_DB (release 2015-01-16)) along with BUSCO (version 5.3.0) were used to assess completeness.

***Assembly quality assessment***

The Merqury.FK tool ( Rhie *et al.*, 2020), run in a Singularity container ( Kurtzer *et al.*, 2017), was used to evaluate assembly quality {for the primary and alternate haplotypes using *k*-mer databases ( *k* = 31) computed prior to genome assembly. The analysis outputs included assembly QV and *k*-mer completeness.

A Hi-C contact map was produced for the final version of the assembly. The Hi-C reads were aligned using bwa-mem2 ( Vasimuddin *et al.*, 2019) and the alignment files were combined using SAMtools ( Danecek *et al.*, 2021). The Hi-C alignments were converted into a contact map using BEDTools ( Quinlan & Hall, 2010) and the Cooler tool suite ( Abdennur & Mirny, 2020). The contact map was visualised in HiGlass ( Kerpedjiev *et al.*, 2018).

The blobtoolkit pipeline is a Nextflow ( Di Tommaso *et al.*, 2017) port of the previous Snakemake Blobtoolkit pipeline ( Challis *et al.*, 2020). It aligns the PacBio reads in SAMtools and minimap2 ( Li, 2018) and generates coverage tracks for regions of fixed size. In parallel, it queries the GoaT database ( Challis *et al.*, 2023) to identify all matching BUSCO lineages to run BUSCO ( Manni *et al.*, 2021). For the three domain-level BUSCO lineages, the pipeline aligns the BUSCO genes to the UniProt Reference Proteomes database ( Bateman *et al.*, 2023) with DIAMOND blastp ( Buchfink *et al.*, 2021). The genome is also divided into chunks according to the density of the BUSCO genes from the closest taxonomic lineage, and each chunk is aligned to the UniProt Reference Proteomes database using DIAMOND blastx. Genome sequences without a hit are chunked using seqtk and aligned to the NT database with blastn ( Altschul *et al.*, 1990). The blobtools suite combines all these outputs into a blobdir for visualisation.

The blobtoolkit pipeline was developed using nf-core tooling ( Ewels *et al.*, 2020) and MultiQC ( Ewels *et al.*, 2016), relying on the Conda package manager, the Bioconda initiative ( Grüning *et al.*, 2018), the Biocontainers infrastructure ( da Veiga Leprevost *et al.*, 2017), as well as the Docker ( Merkel, 2014) and Singularity ( Kurtzer *et al.*, 2017) containerisation solutions.

Table 4 contains a list of relevant software tool versions and sources.

**Table 4.** Software tools: versions and sources.

Software tool	Version	Source
BEDTools	2.30.0	https://github.com/arq5x/bedtools2
BLAST	2.14.0	ftp://ftp.ncbi.nlm.nih.gov/blast/executables/blast+/
BlobToolKit	4.3.7	https://github.com/blobtoolkit/blobtoolkit
BUSCO	5.4.3 and 5.5.0	https://gitlab.com/ezlab/busco
bwa-mem2	2.2.1	https://github.com/bwa-mem2/bwa-mem2
CheckM	1.2.1	https://github.com/Ecogenomics/CheckM
Cooler	0.8.11	https://github.com/open2c/cooler
DIAMOND	2.1.8	https://github.com/bbuchfink/diamond
fasta_windows	0.2.4	https://github.com/tolkit/fasta_windows
FastK	427104ea91c78c3b8b8b49f1a7d6bbeaa869ba1c	https://github.com/thegenemyers/FASTK
GoaT CLI	0.2.5	https://github.com/genomehubs/goat-cli
GTDB-TK	2.3.2	https://github.com/Ecogenomics/GTDBTk
Hifiasm	0.16.1-r375	https://github.com/chhylp123/hifiasm
HiGlass	44086069ee7d4d3f6f3f0012569789ec138f42b84 aa44357826c0b6753eb28de	https://github.com/higlass/higlass
MerquryFK	d00d98157618f4e8d1a9190026b19b471055b22e	https://github.com/thegenemyers/MERQURY.FK
metaMDBG		https://github.com/GaetanBenoitDev/metaMDBG
MitoHiFi	2	https://github.com/marcelauliano/MitoHiFi
MultiQC	1.14, 1.17, and 1.18	https://github.com/MultiQC/MultiQC
NCBI Datasets	15.12.0	https://github.com/ncbi/datasets
Nextflow	23.04.0-5857	https://github.com/nextflow-io/nextflow
PretextView	0.2	https://github.com/wtsi-hpag/PretextView
PROKKA	1.14.5	https://github.com/tseemann/prokka
purge_dups	1.2.3	https://github.com/dfguan/purge_dups
samtools	1.16.1, 1.17, and 1.18	https://github.com/samtools/samtools
Seqtk	1.3	https://github.com/lh3/seqtk
Singularity	3.9.0	https://github.com/sylabs/singularity
TreeVal	1.0.0	https://github.com/sanger-tol/treeval
YaHS	1.1a.2	https://github.com/c-zhou/yahs

### Wellcome Sanger Institute – Legal and Governance

The materials that have contributed to this genome note have been supplied by a Tree of Life collaborator. The Wellcome Sanger Institute employs a process whereby due diligence is carried out proportionate to the nature of the materials themselves, and the circumstances under which they have been/are to be collected and provided for use. The purpose of this is to address and mitigate any potential legal and/or ethical implications of receipt and use of the materials as part of the research project, and to ensure that in doing so we align with best practice wherever possible. The overarching areas of consideration are:

•    Ethical review of provenance and sourcing of the material

•    Legality of collection, transfer and use (national and international)

Each transfer of samples is undertaken according to a Research Collaboration Agreement or Material Transfer Agreement entered into by the Tree of Life collaborator, Genome Research Limited (operating as the Wellcome Sanger Institute) and in some circumstances other Tree of Life collaborators.

## Data Availability

European Nucleotide Archive: *Diplonema japonicum*. Accession number PRJEB62625; https://identifiers.org/ena.embl/PRJEB62625. The genome sequence is released openly for reuse. The *Diplonema japonicum* genome sequencing initiative is part of the Aquation Symbiosis Genomics (ASG) project ( https://www.ebi.ac.uk/ena/browser/view/PRJEB43743). All raw sequence data and the assembly have been deposited in INSDC databases. The genome will be annotated using available RNA-Seq data and presented through the Ensembl pipeline at the European Bioinformatics Institute. Raw data and assembly accession identifiers are reported in Table 1 and Table 2.

## Author information

Members of the Wellcome Sanger Institute Tree of Life Management, Samples and Laboratory Team are listed here: https://doi.org/10.5281/zenodo.10066175.

Members of the Wellcome Sanger Institute Scientific Operations: Sequencing Operations are listed here: https://doi.org/10.5281/zenodo.10043364.

Members of the Wellcome Sanger Institute Tree of Life Core Informatics team are listed here: https://doi.org/10.5281/zenodo.10066637.

Members of the European Bioinformatics Institute ASG Data Portal team are listed here: https://doi.org//10.5281/zenodo.10076466.

Members of the Wellcome Sanger Institute/Aquatic Symbiosis Genomics Project Leadership are listed here: https://doi.org/10.5281/zenodo.10184833.
