# Supplementary material for: The genome sequences of the diplonemid protist Diplonema japonicum YPF1604 and its bacterial endosymbionts Ca. Cytomitobacter primus and Ca. Nesciobacter abundans
Source: Wellcome Open Res. 2026 Sep 21;10:193. Originally published 2025 Apr 14. [Version 3] doi: 10.12688/wellcomeopenres.23917.3 (PMC13598402; doi:10.12688/wellcomeopenres.23917.3)
Supplement: Peer review history

# Reviewer report: Reviewer response for version 3

**DOI:** 10.21956/wellcomeopenres.29840.r169689

**Published:** 2026-09-22

**Authors:** Matthew W Brown

**Affiliations:** 1 Mississippi State University, Starkville, MS, USA

Issues were adequately addressed.

Are sufficient details of methods and materials provided to allow replication by others?

Yes

Is the rationale for creating the dataset(s) clearly described?

Yes

Are the datasets clearly presented in a useable and accessible format?

Yes

Are the protocols appropriate and is the work technically sound?

Yes

Reviewer Expertise:

Protistology, comparative genomics, phylogenomics.

I confirm that I have read this submission and believe that I have an appropriate level of expertise to confirm that it is of an acceptable scientific standard.

# Reviewer report: Reviewer response for version 2

**DOI:** 10.21956/wellcomeopenres.28870.r151977

**Published:** 2026-06-06

**Authors:** Hernan A Lorenzi

**Affiliations:** 1 National Institutes of Health, Bethesda, Maryland, USA

In this Data Note, Tashyreva et al. describe the methods used to generate the genome assembly of the protist *Diplonema japonicum* YPF1604, including its mitochondrial genome and the chromosomal sequences of two bacterial endosymbionts, *Ca. *Cytomitobacter primus and *Ca. *Nesciobacter abundans. The manuscript includes useful links and references to the protocols and tools used for assembly and quality evaluation. The authors also provide numerous assembly statistics and some figures that enable assessment of genome quality. Overall, the information provided will be valuable for the scientific community interested in this organism.

 However, I believe several points should be addressed to improve clarity and provide better insight into the quality and completeness of the genome assembly prior to indexing in *Wellcome Open Research*.

**Major comments**

1. On page 4, the authors report that the final nuclear assembly consists of 121 scaffolds spanning 62.30 Mb, with 118 flagged as “chromosome-level”. Please clarify whether “chromosome-level” implies that each of these scaffolds represents an individual chromosome. If so, how many are flanked by telomeric repeats? Additionally, it would be helpful to briefly state the genome ploidy, the expected nuclear genome size, and the expected number of chromosomes based on methods other than the current sequence.
2. The authors report reference-free quality metrics for the assembly, including k-mer completeness (90.32%) and Quality Value (QV = 59.4). However, these parameters are not explained. Please include a brief description of what these metrics represent (e.g., genome completeness, sequence accuracy, or estimated error rate) and whether the reported values indicate a high-quality assembly.
3. BUSCO analysis indicates that only 30.8% of the euglenozoa_odb10 reference set is present in the assembly. The authors attribute this to poor representation of diplomonads in the dataset, which is a reasonable explanation. However, a more informative approach to assessing completeness would be to compare the *D. japonicum* assembly with the annotated PacBio-based genome of the related species *Diplonema papillatum* (PMID: 37143068). Using approaches similar to BUSCO or tools such as BLASTX, MUMmer, DAGchainer, or ntSynt, the authors could estimate the proportion of conserved genes encoded in *D. japonicum *as a proxy of assembly completeness. Additionally, synteny analysis could help validate scaffold structure and chromosomal boundaries.
4. It would be useful to include a figure summarizing the characteristics of the HiFi long reads, particularly the read length distribution. This would provide insight into the assembly’s ability to resolve long repetitive regions (e.g., tandem duplications or centromeric regions).
5. It is unclear whether the tools used for mitochondrial assembly (MitoHiFi/MitoFinder) are suitable for handling the unusual organization of diplonemid mitochondrial genomes. The new *D. japonicum* mitochondrial assembly consists of 11 DNA fragments, matching the number reported for *D. papillatum*. Although mitochondrial genomes evolve rapidly, it would be valuable to compare these contigs between the two species as an independent validation of assembly accuracy.
6. To identify potential collapsed repetitive regions (e.g., tandem repeats), the authors should consider including a plot showing read depth and coverage along genome scaffolds.
7. Page 6: The section describing RNA extraction appears unrelated to the genome assembly and could be removed.
8. Figures 3, 4, and 5 are not sufficiently discussed in the main text. If these figures are relevant for evaluating assembly quality, their significance should be clearly explained. Otherwise, consider removing them.
9. Figure 5: Please indicate scaffold or contig boundaries on the Hi-C contact map; without these, it is difficult to assess how the Hi-C data support the final scaffold structure.

**Minor comments**

- Page 5: Typo - “AMPure PB beads t”; please revise.
- Page 7: Typo - “Manual curation pf …”; did you mean “Manual curation of …” ?
- Page 7: Typo - “…mitochondrial contig …”; did you mean “…mitochondrial contigs …” ?

Are sufficient details of methods and materials provided to allow replication by others?

Partly

Is the rationale for creating the dataset(s) clearly described?

Partly

Are the datasets clearly presented in a useable and accessible format?

Yes

Are the protocols appropriate and is the work technically sound?

Yes

Reviewer Expertise:

Parasite genomics, Bioinformatics, Microbiome

I confirm that I have read this submission and believe that I have an appropriate level of expertise to confirm that it is of an acceptable scientific standard, however I have significant reservations, as outlined above.

# Reviewer report: Reviewer response for version 2

**DOI:** 10.21956/wellcomeopenres.28870.r151986

**Published:** 2026-04-30

**Authors:** Matthew W Brown

**Affiliations:** 1 Mississippi State University, Starkville, MS, USA

Tashyreva et al. present a very important protistan group, which has been poorly sampled for genome. The paper is presented well and contains expected methods and quality for the field. While the genome appears to be highly contiguous, it does have very poor representation in BUSCO scores, which can be attributed to poor sampling of the group, rapid evolutionary rates which could result in poor hit quality, missing orthologs, poor genome annotation, or a combination of all these. 

 Below are a few comments. 

 - Ideally the authors would present images of each life cycle state as listed in the first paragraph. This could be incorporated as a plate in Figure 1. 

 Figure 2, " A summary of complete, fragmented, duplicated and missing BUSCO genes in the euglenozoa_odb10 set is shown in the top right." Was this BUSCO run simply in nucleotide (genome mode)? I would like to see the BUSCO of overall obd12 and the BUSCO scores following genome gene prediction, which should yield much improved results. 

 - For the assembly of the genome, were reads first removed that map clearly to the endosymbiont or missing bacterial reads (eventhough it is axenic), as well as the MitoGenome,  before the genome was assembled? By doing so the read representation of these taxa would be reduced allowing for a less "meta"-genome assembly. THis can result in better assembly quality. These details are not adequately explained in my view.

Are sufficient details of methods and materials provided to allow replication by others?

Yes

Is the rationale for creating the dataset(s) clearly described?

Yes

Are the datasets clearly presented in a useable and accessible format?

Yes

Are the protocols appropriate and is the work technically sound?

Yes

Reviewer Expertise:

Protistology, comparative genomics, phylogenomics.

I confirm that I have read this submission and believe that I have an appropriate level of expertise to confirm that it is of an acceptable scientific standard, however I have significant reservations, as outlined above.

# Reviewer report: Reviewer response for version 2

**DOI:** 10.21956/wellcomeopenres.28870.r151981

**Published:** 2026-04-07

**Authors:** Francisco Callejas-Hernández

**Affiliations:** 1 Molecular Microbiology and Immunology, Johns Hopkins University Bloomberg School of Public Health (Ringgold ID: 25802), Baltimore, Maryland, USA

The manuscript describes a new genome assembly of Diplonema janonicum, including its mtDNA and a couple of bacterial symbionts.

 The release of a high-quality genome is always scientifically relevant to the research community. However, I identify a few major flaws that need to be addressed to improve the manuscript's readability, interpretation, and reproducibility of the presented results. 

 - First, the material and methods section should be clearly separated from the results. The present form is a combination of both. 

 - Authors should provide as much background as possible about all the genomes described. Has the genome size of D. japonicum been estimated (including mtDNA)? How many chromosomes are expected (on this or related species)?. That could help the authors support their findings regarding the completeness and fragmentation levels, especially in the absence of a curated set of BUSCO references for this species.

 I agree with the author's hypothesis for the low BUSCO score, and, as in this case, it is not informative, authors should consider removing it. 

 - Authors should provide the completeness per haplotype, not combined.

 - What about the genome annotation (nuclear)? Is the gene count aligning with the expected results for these species?

 - I consider Table 1 to contain redundant information. NCBI accessions have already been provided in the "Data availability section", so they should remove it from the table. The same for sequencing information. The sequencing coverage can be presented in the main text or in supplementary tables. Additional useful assembly information can be added instead. For instance, the number of gaps (from the scaffolding), the gap total length, L50, L90, and especially the assembly stats of the mitochondrial genome. 

 The information in Table 3 has already been provided in the "Data availability section", so the authors should consider removing it from the main text. 

 - Next, I consider the major flaw of the paper is the assembly methodology, which affects Figures 3 and 4. Did the authors assemble all the reads in a single run (meta-assembly)? If my interpretation is correct, and although this is a common practice in some circumstances, wouldn't it be better to classify reads first and produce independent assemblies (bacterial and protist)? That could reduce the risk of chimeric assemblies and potentially lower fragmentation levels in the final assemblies. 

 In addition, could the authors please specify what manual curation of the assembly entails?  

 - I suggest moving Table 2 as supplementary material, as it does not provide relevant information to discuss in the main text. 

 - I consider Table 4 unnecessary for the main text. Authors should instead provide proper citations in the materials and methods section.

Are sufficient details of methods and materials provided to allow replication by others?

Partly

Is the rationale for creating the dataset(s) clearly described?

Partly

Are the datasets clearly presented in a useable and accessible format?

Partly

Are the protocols appropriate and is the work technically sound?

Partly

Reviewer Expertise:

Parasite genomics; Transcriptomics; Gene expression; Bioinformatic tools for molecular biology.

I confirm that I have read this submission and believe that I have an appropriate level of expertise to confirm that it is of an acceptable scientific standard, however I have significant reservations, as outlined above.

# Reviewer report: Reviewer response for version 1

**DOI:** 10.21956/wellcomeopenres.26386.r122827

**Published:** 2025-05-30

**Authors:** Tsvetan Bachvaroff

**Affiliations:** 1 University of Maryland Center for Environmental Science, Baltimore, MD, USA

The authors present a genome assembly of the 'classic' diplonemid Diplonema japonicum along with two bacteria and several mitochondrial contigs using pacbio reads.  The diplonemids s.l. are an incredibly abundant lineage in marine systems and are enigmatic making this genome announcement a significant step.  Even within the constraints of a brief report this announcement could be considerably improved by adding in layers of important biological detail about the results, making the work more coherent, and adding important details.  The analysis pipeline does not seem well tailored for the group and there are discordant elements in the figures.  Some digressive methods elements could also be removed, especially if the analysis pipeline is publicly available and standardized.

 None of these criticisms are meant to deter the overall mission of making previously obscure and likely globally important diplonemid genomes available, nor to critique the sequencing facility, culturing efforts, or authors.  Probably the analysis pipeline just needs a minor amount of adjustment to produce a better product.  

 Introduction: Provide a brief summary of the organism's importance, mode of nutrition (and what the nutrients are).  This does not need to be extensive but should be as rich in details as the different life stages which are clearly described.  With a few sentences the overall picture of how the cell is cultured, growth rate and ultimate yield as well as other features should be quickly presented here or in the results.

 The meta-assembly.  Three different categories were assembled individually -- 1) nuclear, 2) mitochondrial, 3) the bacteria -- but no information is provided on how reads were selected or partitioned to be sent to the different assemblers.  Many assemblers can quickly assemble metagenomic datasets that include organelles, bacteria, and nuclear contigs, but the approach in this instance is unclear.  Were all the data provided to each assembly tool? If so, why? Or were reads sorted into categories from a primary meta-assembly? What is the justification for using these different tools?  

 The presentation of contig and scaffold information in the text and figures is redundant and unclear. e.g. Figure 2 seems to be contig-based and is largely redundant with other figures. For clarity the scaffolds alone could be considered the final result.  

 A typical reader might ask: Why so many chromosomes?  Is this an artifact? What do the telomeres or subtelomeric regions look like?  What sort of repeats are found in the genome? 

 Presumably not every one of the 121 -- maybe 118 sequences are considered to be full chromosomes, and some simple methods could be used to winnow or refine this selection (based on size, gene content, RNAseq mapping or other criteria).   

 Maybe this high chromosome number is a standard feature of the group or perhaps there are more joins that could be made.  Other assembly methods that are less centered on haplotigs than hifiasm might provide better starting contigs or at least point the way to consistent and inconsistent contigs by comparing assembly methods. Probably a more useful figure than 2, and 4 would be a schematic of a single chromosome with repeats, genes and other features mapped on to it.   

 Figure 3 probably should have the colors removed or based on a different criterion.  At first glance it seems to indicate that there are 3 chordate contigs with a span of 1.5M within the assembly!  This seems incongruous.  The methods for creating the blob plot are quite detailed and yet no interpretation or presentation of the results is provided in the text. Diamond is so fast that diamond blastx can be quickly run vs nr based on all predicted genes -- the reliance on uniprot may have been a problem here.  Many researchers also use custom, lineage specific datasets (for example using all available related genomes even if few are available) for gene prediction and contig -> taxonomy assignment.  In addition, the two bacteria, mitochondrial contigs and any other features of the assembly should be included on the blob figure and marked with a different color or label.  The y axis is an estimate of coverage and needs to be correctly labeled.  The suspicious >>1k depth contig should be carefully checked over.  Similarly, figure 4 should not be colored by taxonomy and could be deleted unless better methods are applied to make and confirm these assignments.  

 Gene prediction / RNAseq

 A repeat prediction & assessment paragraph should be placed before the gene prediction paragraph. Please provide information on the RNAseq or gene prediction which appears to have not worked very well based on the low busco score (60/130 missing). Typically, a de novo RNAseq transcriptome assembly can be used to tabulate busco scores in parallel and point out potential gene prediction problems. Presumably there are some features to the genes in this organism that defy simple annotation but at least some information on this topic should be provided.

 The paragraph beginning "The primary assembly..." is not correctly phrased and should read, "Gene prediction based on (sketch pipeline details in a phrase or two) showed a BUSCO..." and should include the total number of predicted genes and some parameters such as average intron exon counts, intron exon boundaries and other standard information.  

 Organellar genome

 Please provide information for the mitochondrial contigs.  These contigs could be clearly marked on the blob plot and described in a few sentences to summarize these results (N50, the total span, GC/AT bias, number of contigs, coverage, predicted genes, scrambling, editing or any other information).  This is one of the more interesting features of the group and merits more detail.  The sentence starting with "The mitochondrial genome was also assembled ... " is phrased as if there was a single contig.  Given the unusual nature of this organelle the assembly and annotation methods might need adjustment.  

 Table 3 / Figure 6: Is this an updated bacterial genome assembly from the previously published versions?  Are these single contig genomes?  What is the coverage, GC, gene content, checkM, gtdb taxonomy, and other details of these genomes?  

 Other details:

 There is an inconsistent use of italics for the bacterial names.

 Statements like "The cumulative assembly plot shows curves ..." could be replaced with informative results statements ending with reference to the figures.    

 The kmer completeness in the text is presented as 100% in the table and 90.32% in the text.  

 The statement that Diplonema was grown 'axenically' in the methods is not consistent with the results.

 The taxonomic assignment is assigned to a specific person identified in Methods, but this is the first author which is redundant.

 In the methods are the cells ‘dissected’? 

 Underscores need to be removed from figures. In general, the text needs to be cleaned up in all the figures.

Are sufficient details of methods and materials provided to allow replication by others?

Yes

Is the rationale for creating the dataset(s) clearly described?

Yes

Are the datasets clearly presented in a useable and accessible format?

No

Are the protocols appropriate and is the work technically sound?

No

Reviewer Expertise:

Dinoflagellate genomics and gene family evolution.

I confirm that I have read this submission and believe that I have an appropriate level of expertise to confirm that it is of an acceptable scientific standard, however I have significant reservations, as outlined above.

# Reviewer report: Reviewer response for version 1

**DOI:** 10.21956/wellcomeopenres.26386.r122358

**Published:** 2025-05-19

**Authors:** Mahwash Jamy

**Affiliations:** 1 Swedish University of Agricultural Sciences, Uppsala, Sweden

Tashyreva et al. presented a high-quality genome of Diplonema japonicum and its bacterial endosymbionts, valuable for studying diplonemid-bacterial symbioses. I have a few minor comments:

 1. As diplonemids are known to possess bizarre mitochondria (e.g.  

 refer to 1), I would like to see some more information about the mitochondrial genome. How many genes? How large was the mitochondrial genome? Perhaps the authors could add a figure showing the genome map of the mitochondria similar to Fig 6. 

 2. Methods: How much material was used for nucleic acid extraction? It would be good to have a rough idea of the size/weight of the pellet, or an estimate of the number of cells. How long were the diplonemid cells grown in culture?

Are sufficient details of methods and materials provided to allow replication by others?

Partly

Is the rationale for creating the dataset(s) clearly described?

Yes

Are the datasets clearly presented in a useable and accessible format?

Yes

Are the protocols appropriate and is the work technically sound?

Yes

Reviewer Expertise:

Protistology, phylogenetics, metabarcoding, environmental sequencing, genomics

I confirm that I have read this submission and believe that I have an appropriate level of expertise to confirm that it is of an acceptable scientific standard, however I have significant reservations, as outlined above.

## References

[1] : Massive mitochondrial DNA content in diplonemid and kinetoplastid protists. *IUBMB Life* .2018;70(12) : 10.1002/iub.18941267-1274 30291814 10.1002/iub.1894 PMC6334171
